# Design of novel pyrazole and benzofuran-based derivatives as potent acetylcholinesterase inhibitors for Alzheimer’s disease management

**DOI:** 10.3389/fchem.2025.1614462

**Published:** 2025-05-22

**Authors:** Mohamed El Fadili, Amine Ez-Zoubi, Mourad Aloui, Somdutt Mujwar, Hatem A. Abuelizz, Menana Elhalaoui, Adnan Amin

**Affiliations:** ^1^ LIMAS Laboratory, Faculty of Sciences Dhar El Mahraz, Sidi Mohamed Ben Abdellah University, Fez, Morocco; ^2^ Laboratory of Applied Organic Chemistry, Faculty of Sciences and Techniques, Sidi Mohamed Ben Abdellah University, Route d’Imouzzer, Fez, Morocco; ^3^ Chitkara College of Pharmacy, Chitkara University, Rajpura, Punjab, India; ^4^ Department of Pharmaceutical Chemistry, College of Pharmacy, King Saud University, Riyadh, Saudi Arabia; ^5^ Department of Life Sciences, Yeungnam University, Gyeongsan, Republic of Korea

**Keywords:** Alzheimer’s disease, AChE, 3D-QSAR, DFT, molecular docking, molecular dynamics

## Abstract

**Introduction:**

Being a complex neurodegenerative disease with many clinical features, Alzheimer’s disease calls for multiple-targeted drugs to treat several aspects of its progression in the human body. The present study sheds light on evaluating and designing novel pyrazole and benzofuran-based derivatives as potent acetylcholinesterase (AChE) inhibitors with improved antioxidant features to manage Alzheimer’s disease.

**Materials:**

Various molecular interaction fields, specifically steric, electrostatic, hydrophobic, acceptor, and donor fields of hydrogen bonds, were examined using 3D-QSAR models to predict inhibitory activity against the AChE enzyme, which was successfully validated through both external and internal assessments.

**Results and discussion:**

Consequently, the CoMFA and CoMSIA/ SEHDA models led to the design of the candidate compound C27** as one of the most potent acetylcholinesterase inhibitors while building on the most active molecule (C7). Both C27** and C7 revealed their significant chemical reactivity after their optimization with B3LYP 6-31G (d, p) using the density functional theory (DFT), in addition to large similarities to the candidate drugs with desired pharmacokinetic and physicochemical features and good levels of molecular stability towards the crystal structure of human acetylcholinesterase protein (PDB ID of 4EY7).

## 1 Introduction

More than 50 million people around the world are suffering from Alzheimer’s disease (AD), a complex and progressive neurodegenerative disorder that disrupts the biological functions of the central nervous system (CNS) (Guo et al., 2020). Until now, Alzheimer’s remains a chronic and potentially incurable disease despite serious and ongoing efforts to look for more effective drugs that would gradually improve the patient’s memory, restore his multiple cognitive and linguistic abilities, avoid complete disability, and ultimately save a large segment of the worldwide population from the potentially devastating consequences that may result from this neurodegenerative disorder (Besli et al., 2025). Alzheimer’s disease can be medically, psychologically, and socially treated in two main ways. Firstly, pharmaceutical treatments that act as acetylcholinesterase inhibitors, including Donepezil, Rivastigmine, and Galantamine, increase the levels of neurotransmitters, such as acetylcholine, involved in memory and learning (Goschorska et al., 2018; Gupta et al., 2022). Second, N-methyl-D-aspartate (NMDA) receptor antagonists such as Memantine, a drug that regulates glutamate activity as another neurotransmitter, may be useful in the moderate to severe stages of the disease (Almutairi et al., 2024; Guler et al., 2024). However, like other medications, Memantine, Donepezil, and other inhibitors may cause harmful effects such as neurological and psychological effects (dizziness, confusion, hallucinations, irritability, excessive drowsiness, headache, insomnia, and depression). Cardiovascular effects (high blood pressure and increased heart rate), gastrointestinal effects (nausea, vomiting, and constipation), as well as effects on the musculoskeletal system, fatigue, loss of appetite, and skin allergic reactions (Nagakura et al., 2013; Yamada et al., 2005). Due to its complexity, AD requires multiple-targeted drugs to treat various aspects of its progression in the human body, avoiding some of its undesirable side effects. A recent study authored by Besli et al. (2025) has synthesized a range of novel multi-target compounds acting as dual acetylcholinesterase (AChE) and butyrylcholinesterase (BuChE) inhibitors with complementary antioxidant functions, to be suggested as potential therapeutics for Alzheimer’s disease management, in which deep *in silico* investigations were carried out to evaluate the efficacy of these novel pyrazole and benzofuran-based derivatives, which were sufficiently examined using both *in vitro* and *in vivo* assays. The three-dimensional structure-activity relationship (SAR) was first analyzed using the best classical Comparative Molecular Field Analysis (CoMFA) and best Comparative Molecular Similarity Index Analysis (CoMSIA) models, which examine different molecular interaction fields (El fadili et al., 2022a; El fadili et al., 2023a). In principle, the CoMFA model considers steric and electrostatic fields, while the CoMSIA model extends this analysis by also incorporating hydrophobic, hydrogen bond acceptor, and hydrogen bond donor fields. Both the quantitative 3D-SAR models were properly tested using external and internal validations, to explore the favorable and unfavorable areas of anticholinesterase inhibitory activity, and eventually designing novel candidate’ drugs, to be optimized using density functional theory (DFT) as a crucial tool in computational chemistry and materials science for predicting reaction mechanisms and analyzing the electronic properties of optimized molecules with accurate energies of frontier molecular orbitals (FMOs). It offers greater accuracy than the Hartree–Fock (HF) method for ground-state properties, while maintaining a similar or only slightly higher computational cost. These advantages make DFT the method of choice for many theoretical studies in chemistry, physics, and materials science (Hamad et al., 2023). For more accuracy, improved electron density representation, better modeling of non-covalent interactions, and reliable geometry optimization, DFT calculations were performed using the 6-31G (d,p) basis set, a versatile and dependable choice, especially when balancing precision and computational cost, rather than alternative basis sets such as STO-3G, 3-21G, or 6-31G without d and p polarization functions (El fadili et al., 2023b). The optimized compounds were subsequently approved by *in silico* predictions of pharmacokinetics profiling (Nouioura et al., 2025), and subjected to drug-likeness based on Lipinski, Veber, Egan, Muegge, and Ghose rules (Aloui et al., 2024a; El fadili et al., 2023c). Then, the molecular docking simulations have been conducted for these optimized compounds against the 3D crystal structures of human Acetylcholinesterase (PDB ID of 4EY7). Finally, the resulting protein-ligand complexes have been analyzed through molecular dynamics (MD) simulations over a 100-nanosecond timeframe (Ed-Dahmani et al., 2025; Ez‐zoubi et al., 2025).

## 2 Materials and methods

### 2.1 Experimental database

Two innovative series of chemical products: benzofuran-derived Donepezil equivalents (C1-9) and their pyrazole-based analogs (C10-18), engineered as promising dual inhibitors of cholinesterase enzymes with enhanced antioxidant characteristics, targeting the simultaneous treatment of several pathological aspects of Alzheimer’s disease, as presented in Table 1, in which the chemical compound labeled C7 was the most effective anti-acetylcholinesterase inhibitor (AChE IC_50_ of 0.39 μg/mL) (Elkotamy et al., 2025).

**TABLE 1:** AChE IC_50_ inhibitory activities for novel pyrazole and benzofuran-based derivatives.

N°	Structure	Ar_1_	Ar_2_	AChE IC_50_ (µg/mL)
C1	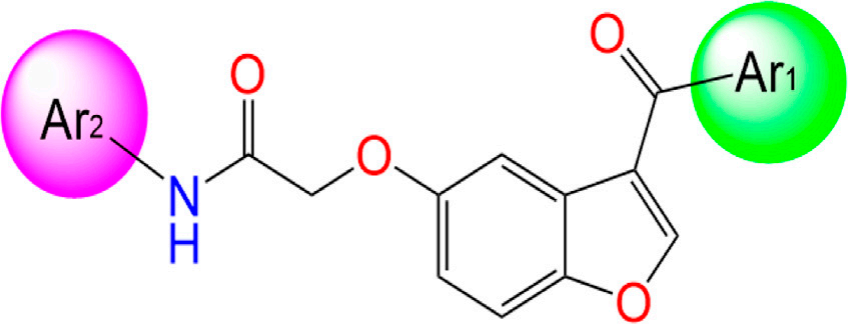	Ph	Ph	0.77
C2		Ph	4-Me-C_6_H_4_	8.26
C3		Ph	4-Cl-C_6_H_4_	16.46
C4		4-Me-C_6_H_4_	Ph	1.24
C5		4-Me-C_6_H_4_	4-Me-C_6_H_4_	23.67
C6		4-Me-C_6_H_4_	4-Cl-C_6_H_4_	21.82
C7		4-Cl-C_6_H_4_	Ph	0.39
C8		4-Cl-C_6_H_4_	4-Me-C_6_H_4_	5.01
C9		4-Cl-C_6_H_4_	4-Cl-C_6_H_4_	7.96
C10	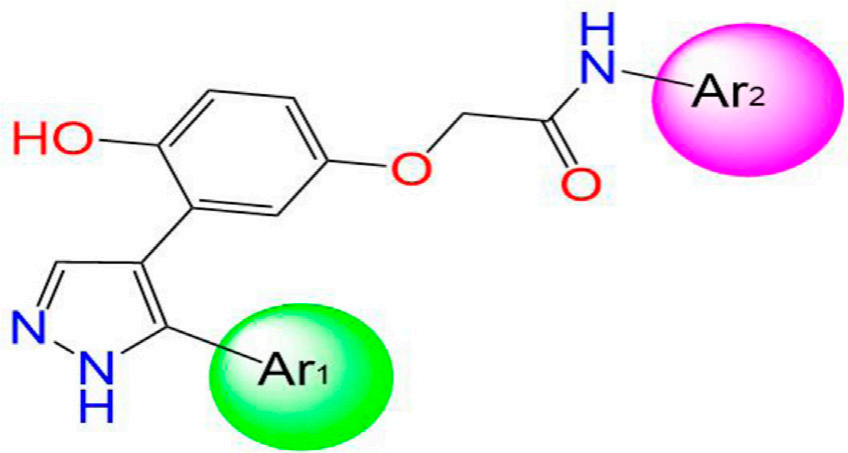	Ph	Ph	19.35
C11		Ph	4-Me-C_6_H_4_	11.7
C12		Ph	4-Cl-C_6_H_4_	17
C13		4-Me-C_6_H_4_	Ph	16.3
C14		4-Me-C_6_H_4_	4-Me-C_6_H_4_	1.97
C15		4-Me-C_6_H_4_	4-Cl-C_6_H_4_	1.24
C16		4-Cl-C_6_H_4_	Ph	5.75
C17		4-Cl-C_6_H_4_	4-Me-C_6_H_4_	18.81
C18		4-Cl-C_6_H_4_	4-Cl-C_6_H_4_	54.01

### 2.2 3D-QSAR study

**FIGURE 1:**
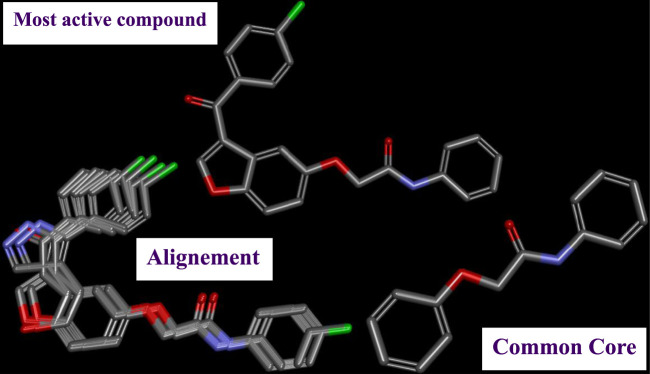
2D structures of the synthesized compound with the most anti-acetylcholinesterase activity (C7), the superposition or alignment result for both pyrazole and benzofuran-based derivatives on C7, and the Common core.

Three-dimensional quantitative relationships between the chemical structures of the new pyrazole- and benzofuran-based derivatives towards anti-acetylcholinesterase activities were established using SYBYL-X 2.0 software, where the partial least square (PLS) method was properly employed to generate the classical CoMFA model and all the possible CoMSIA models responsible for the studied inhibitory activity, after several randomizations performed by dividing the experimental data into two subsets: A training set with 70% of the total data and a test set including 30% of the whole data. The CoMFA model focuses primarily on steric and electrostatic molecular fields, while the CoMSIA models additionally involve hydrophobic, acceptor, and donor of hydrogen bonds’ fields. The optimal 3D-QSAR model was selected for external validation using a test set comprising the five chemical compounds C2*, C10*, C14*, C15*, and C16*. The remaining thirteen molecules were chosen to develop both CoMFA and CoMSIA models. Among them, the compound C7, belonging to the pyrazole family, exhibited the most potent anti-acetylcholinesterase activity. The chemical structures of the active molecules were initially optimized by minimizing their energy (in kcal/mol) to ensure the stability of each geometric conformation. This process involved assigning Gasteiger-Hückel charges and setting the convergence parameter of the Powell-gradient algorithm to 0.001 kcal/(mol·Å), with a maximum of 10,000 iterations. The optimization was carried out using the Tripos force field. Superposition analysis identified 2-phenoxy-N-phenylacetamide as the common core structure aligning both pyrazole- and benzofuran-based derivatives, as illustrated in Figure 1.

### 2.3 Drug-likeness and ADMET *in silico* predictions

One of the key challenges in modern pharmaceutical research is to develop safe and highly effective drugs while optimizing time, effort, and costs. To achieve this, computer-aided drug design (CADD) has become an essential tool in the field (Besli et al., 2025). At this stage of development, the pharmacokinetic properties of the designed compounds-such as absorption, distribution, metabolism, excretion, and toxicity-were accurately predicted using the Swiss ADME and PKCSM online platforms (Nouioura et al., 2024a; Nouioura et al., 2024b).

### 2.4 DFT calculation studies

Density functional theory (DFT) is a fundamental computational approach in quantum chemistry, enabling detailed studies of molecular structures, electronic properties, and stability. In this study, DFT calculations were performed to optimize the molecular geometries of two substituted pyrazole derivatives (C7 and C27**). Computational analysis was carried out using the hybrid function B3LYP in conjunction with the 6-31G (d, p) basis set for initial geometry optimization, followed by refinement with the more extended 6-31G (d, p) basis set as a split divalent zeta reference with d-type polarization functions on heavy atoms and p-type on hydrogen atoms, to allow distortion of the electron cloud, thus improving the accuracy of geometries and calculated energies. To confirm that the optimized structures represent the true minimum on the potential energy surface, vibrational frequency analyses were performed, and convergence was verified in all cases with no spurious frequencies, confirming that the geometries are true fixed points (Ez-zoubi et al., 2025). All calculations were performed using Gaussian 09W software, guaranteeing reliable results based on well-established quantum mechanical principles. Subsequently, the optimized structures were visualized and analyzed using GaussView 6 to facilitate interpretation of molecular conformations and electronic distribution. This approach provides valuable information on the stability and electronic characteristics of the compounds studied, enabling further experimental and theoretical investigations (Mermer et al., 2020; Nimr Ajeel et al., 2017).

### 2.5 Molecular docking simulations

Molecular docking simulations were performed using Autodock 4.2 and Discovery Studio 2021 software (Ed‐Dahmani et al., 2024; Seddoqi et al., 2024). Two chemical compounds (C7 and C27**), along with the most active compounds, were selected for docking simulations against the 3D crystal structure of human Acetylcholinesterase (PDB ID of 4EY7). To prepare the targeted protein, Gasteiger charges were added, water (H_2_O) molecules were removed, and all suspended and co-crystallized ligands were eliminated (El fadili et al., 2022b; Aloui et al., 2024b; Bouzammit et al., 2024a). Subsequently, the designed compounds were docked to the processed protein using Autodock 4.2 software (Aloui et al., 2024a; Bouzammit et al., 2024b; Ed-Dahmani et al., 2024a). The docking grid boxes were consistently centered on the target AchE protein (4EY7) with Cartesian coordinates set at x = −2.95, y = −40.107, and z = 30.729, using a default spacing of 0.375 angstroms. In the final stage, the Discovery Studio 2021 software was utilized to visualize the resulting intermolecular interactions in both two-dimensional and three-dimensional representations (Assaggaf et al., 2023; Ed-Dahmani et al., 2024b; El fadili et al., 2023d).

### 2.6 Molecular dynamics simulations

Molecular dynamics simulation was performed by using the Desmond program of Schrödinger’s Maestro software for 100 ns timeframe to examine the thermodynamic stability of the produced intermolecular contacts throughout the simulation time. The pyrazole-based designed ligands C7 and C27** complexed with AChE enzyme, were shortlisted based upon 3D-QSAR modelling and the observed docking results. The output complex files generated after molecular docking were chosen as input files for the execution of molecular dynamics (Shinu et al., 2022). The MD simulation was performed by employing the OPLS_2005 force field, as it has been rigorously parameterized and validated for proteins, nucleic acids, and small molecules. The macromolecular complex structures were preprocessed for executing MD simulation by assigning bond orders by referring CCD database, addition of missing hydrogens, creating disulfide bonds, and zero zero-order bond to metals, followed by their model optimization by using PROPKA at pH 7.0 (Kciuk et al., 2023). Additionally, its optimized water models ensure proper solvation and realistic molecular behavior in an aqueous environment, along with efficient energy calculations and system stability during simulations, making it highly suitable for protein-ligand simulations. The total simulation time was 100 ns at 1.2 energy and approximately 1000 frames. Nose-Hoover chain method-based thermostat was used to maintain the system temperature of 330 K and Martyna-Tobias-Klein method based Barostat was employed to maintain the pressure condition at 1 atm by considering relaxation time of 2.0 ps and isotropic coupling style, followed by neutralization of the system by using counter ions by 0.15 M salt (Na+, Cl) (Mujwar and Harwansh, 2022; Fidan et al., 2023; El fadili et al., 2024).

## 3 Results and discussions

### 3.1 3D-QSAR study

**FIGURE 2:**
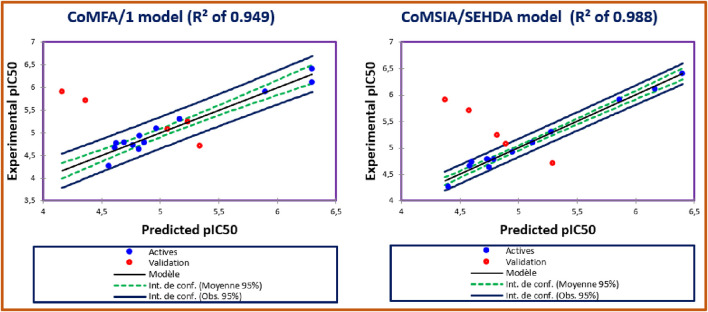
Correlation between the experimental and predicted AChE-antagonizing activities using CoMFA/1 and CoMSIA/SEHDA models, respectively.

Using PLS regression, seventeen 3D/QSAR models were successfully developed to predict AChE IC_50_ inhibitory activities of novel pyrazole and benzofuran-based analogs, as outlined in Table 2. These predictive models were constructed by evaluating various molecular interaction fields, including Steric (S), Electrostatic (E), Hydrophobic (H), as well as Hydrogen bond Donor (D) and Acceptor (A) fields. Typically, the Steric and Electrostatic fields are fundamental components of the CoMFA model, whereas the CoMSIA models incorporate all five fields: Steric, Electrostatic, Hydrophobic, Donor, and Acceptor of H bonds. To ensure the reliability and predictive power of the 3D-QSAR models, several statistical parameters were considered. The cross-validated determination coefficient (Q^2^cv), calculated using the leave-one-out (LOO) method, should be greater than 0.6, while the non-cross-validated determination coefficient (*R* ^2^) obtained through a non-cross-validation procedure must exceed 0.7. Additionally, the Fisher value (F) should surpass Fisher’s critical value, and the standard estimation error (SEE) should be minimized for an optimal number of principal components (ONC). Moreover, the external validation determination coefficient must be greater than 0.6. These statistical parameters were computed for the CoMFA model and sixteen possible CoMSIA models, all of which were generated using the same training set consisting of thirteen molecules. Each model underwent internal validation through the CV-LOO method and external validation using a predetermined test set (C2*, C10*, C14*, C15*, and C16*). The obtained results demonstrate that the CoMFA (*R* ^2^ = 0.949, Q^2^_cv_ = 0.797, R^2^_pred_ = 0.888, SEE = 0.169, ONC = 3), and CoMSIA/SEHDA (*R* ^2^ = 0.988, Q^2^_cv_ = 0.825, R^2^_pred_ = 0.972, SEE = 0.125, ONC = 8) were the best 3D/QSAR models to predict AChE inhibitory activity, as displayed in Table 2. Both selected models were sufficiently validated using both internal and external validation with the highest correlation coefficients, revealing that steric, electrostatic, hydrophobic, acceptor, and donor of Hydrogen bonds have a key function in both inhibitory activities, where the predicted AChE activities for the training and test set molecules are given in Table 3. The quantitative correlations between the experimental and predicted AchE inhibitory activities for CoMFA and CoMSIA/SEHDA are presented in Figure 2, in which the training and test sets’ molecules were marked in blue and red, respectively.

**TABLE 2:** Statistical outcomes of 3D-QSAR models across different molecular field combinations for predicting AChE IC_50_ inhibitory activity.

All possible 3D-QSAR Models	Fractions of molecular interactions fields					*Q* ^*2*^_*cv*_	*R* ^*2*^	*SEE*	*F*	*ONC*	*R* ^*2*^ *pr*
	Steric (S)	Electrostatic (E)	Hydrophobic (H)	Donor (D)	Acceptor (A)						
COMFA	0.542	0.458	-	-	-	0.797	0.949	0.169	55.609	3	0.888
CoMSIA/SEA	0.079	0.815	-	-	0.105	0.793	0.988	0.121	42.433	8	0.732
CoMSIA/SEH	0.041	0.579	0.380	-	-	0.811	0.975	0.158	28.016	7	0.930
CoMSIA/SED	0.080	0.828	-	0.092	-	0.775	0.988	0.121	42.581	8	0.732
CoMSIA/SHD	0.060	-	0.558	0.382	-	0.762	0.975	0.158	27.916	7	0.952
CoMSIA/SHA	0.059	-	0.533	-	0.408	0.822	0.975	0.159	27.424	7	0.946
CoMSIA/SDA	0.256	-	-	0.275	0.468	0.788	0.938	0.210	21.263	5	0.863
CoMSIA/EHA	-	0.485	0.378	-	0.138	0.818	0.988	0.122	41.828	8	0.964
CoMSIA/EHD	-	0.496	0.388	0.116	-	0.802	0.988	0.122	41.896	8	0.965
CoMSIA/EDA	-	0.865	-	0.055	0.081	0.799	0.988	0.108	60.261	7	0.734
CoMSIA/HDA	-	-	0.568	0.167	0.265	0.787	0.974	0.161	26.952	7	0.876
CoMSIA/EHDA	-	0.454	0.368	0.074	0.104	0.810	0.988	0.122	41.555	8	0.964
CoMSIA/SHDA	0.057	-	0.489	0.174	0.280	0.800	0.974	0.160	27.227	7	0.945
CoMSIA/SEDA	0.034	0.644	-	0.143	0.178	0.784	0.988	0.121	41.994	8	0.736
CoMSIA/SEHA	0.039	0.466	0.367	-	0.128	0.821	0.988	0.124	40.236	8	0.971
CoMSIA/SEHD	0.028	0.433	0.293	0.245	-	0.806	0.973	0.163	26.060	7	0.896
CoMSIA/SEHDA	0.038	0.435	0.357	0.072	0.097	0.825	0.988	0.125	39.863	8	0.972
	Total fractions of 1					External and internal validations					

Abbreviations: *Q* ^*2*^_*cv*_: the cross-validation determination coefficient, *R* ^*2*^: the non-cross-validation determination coefficient, *SEE*: the standard estimation error, *F*: the Fischer test value, *ONC*: the optimum number of components, and *R* ^*2*^ *pr*: the external validation determination coefficient.

**TABLE 3:** Prediction of AChE-antagonizing activities using CoMFA and CoMSIA/SEHDA models for both training and test sets.

N°	AChE IC_50_ (µg/mL)	pIC_50_	Predicted pIC_50_ using CoMFA model	Predicted pIC_50_ using CoMSIA/SEHDA model
C1	0.77	6.11	6.295	6.166
C3	16.46	4.78	4.862	4.731
C4	1.24	5.91	5.895	5.862
C5	23.67	4.63	4.818	4.751
C6	21.82	4.66	4.615	4.586
C7	0.39	6.41	6.297	6.401
C8	5.01	5.3	5.169	5.283
C9	7.96	5.1	4.966	5.119
C11	11.7	4.93	4.824	4.950
C12	17	4.77	4.624	4.744
C13	16.3	4.79	4.694	4.791
C17	18.81	4.73	4.765	4.605
C18	54.01	4.27	4.565	4.400
C2*	8.26	5.08	5.065	4.896
C10*	19.35	4.71	5.336	5.290
C14*	1.97	5.71	4.362	4.580
C15*	1.24	5.91	4.161	4.374
C16*	5.75	5.24	5.237	4.817

### 3.2 A graphical analysis of validated 3D-QSAR models

**FIGURE 3:**
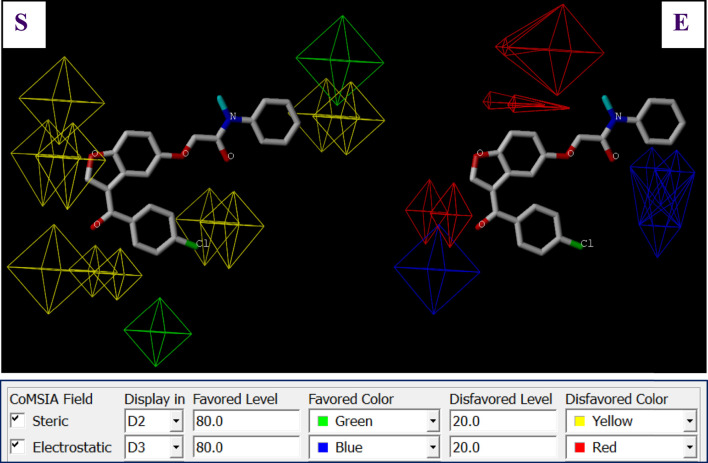
Steric (S) and electrostatic (E) molecular fields implicated in the CoMFA-1 model for C7.

**FIGURE 4:**
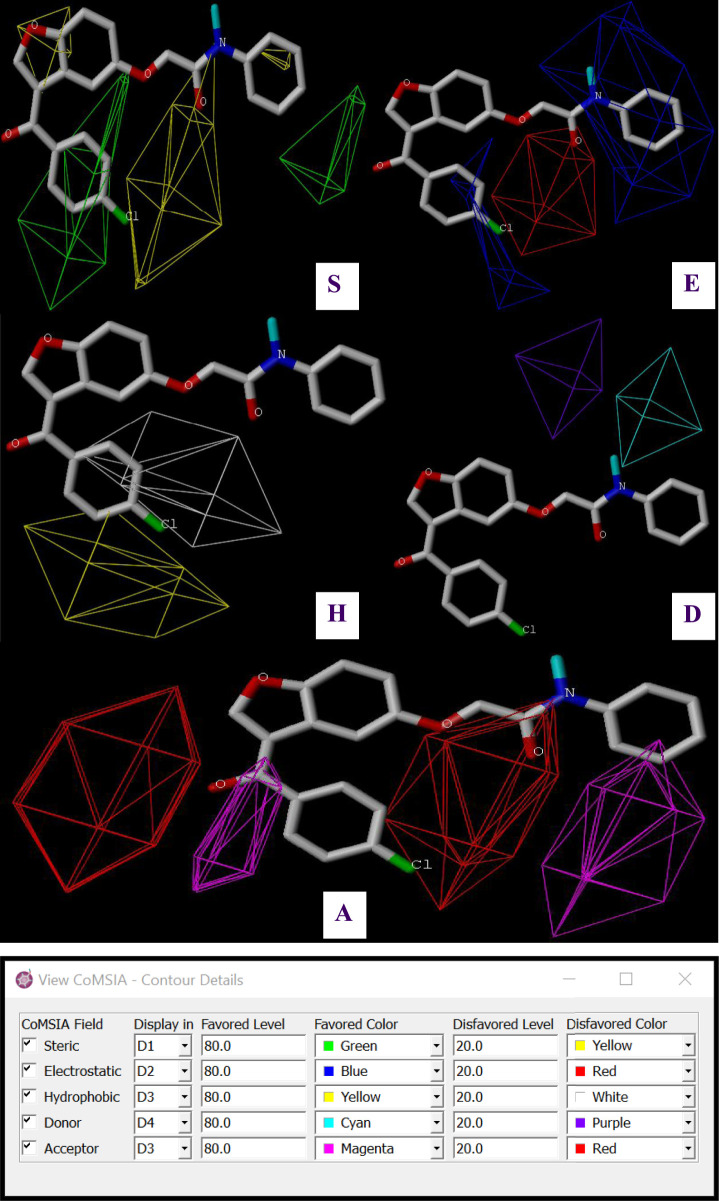
Steric (S), Electrostatic (E), Hydrophobic (H), Acceptor (A) and Donor (D) molecular interaction fields predicted by CoMSIA/SEHDA model for C7.

**FIGURE 5:**
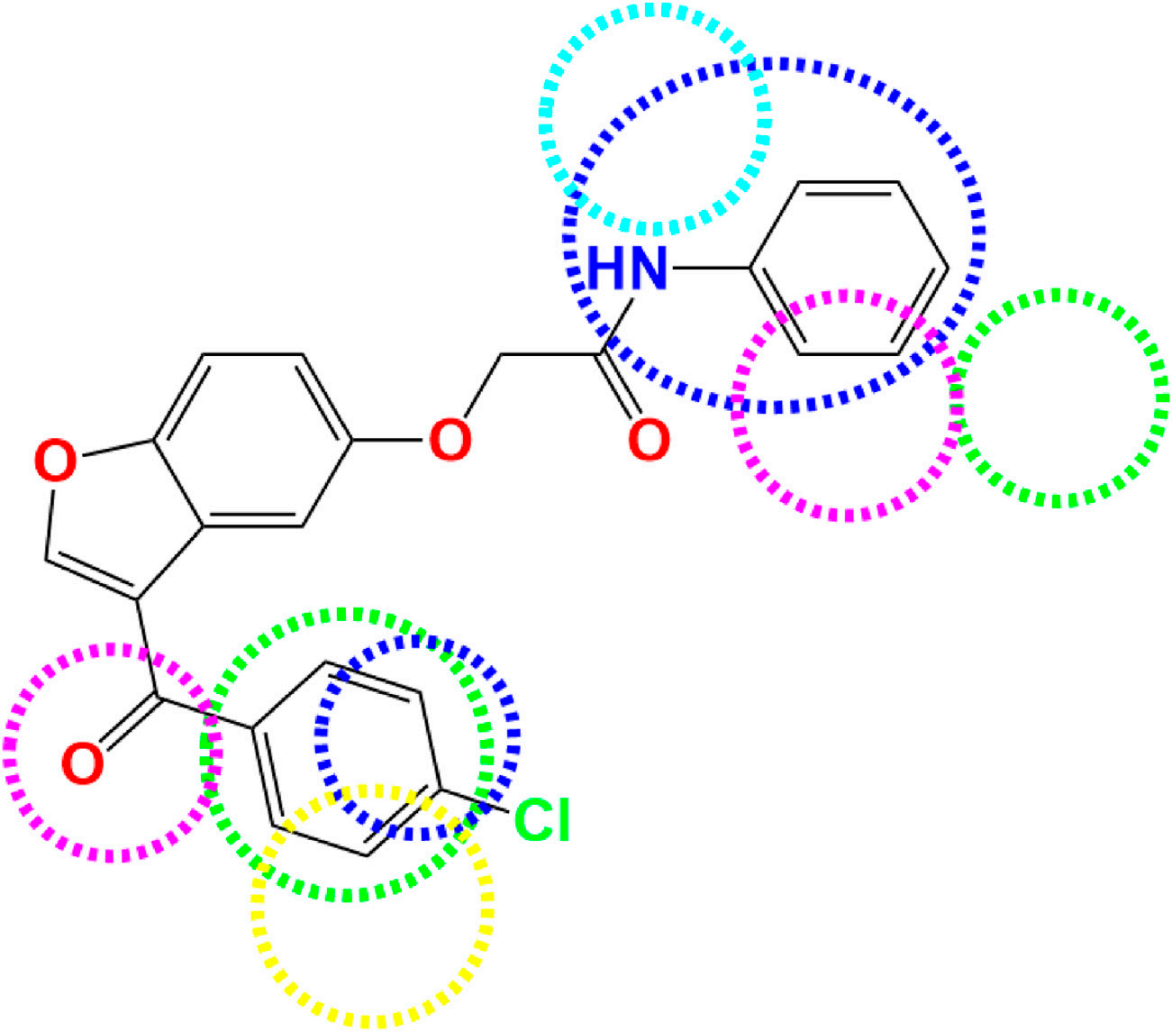
Favorable regions of various molecular fields derived from 3D-QSAR models: Steric (in green), Electrostatic (in blue), Hydrophobic (in yellow), Acceptor (in magenta), and Donor of hydrogen bonds (in cyan).

Three-dimensional contour maps of molecular interaction fields, as analyzed through CoMFA and CoMSIA/SEHDA models, were effectively utilized to identify the key regions influencing steric, electrostatic, hydrophobic, and hydrogen bond donor/acceptor interactions, which play a role in the AChE inhibitory activity of pyrazole- and benzofuran-based derivatives. The results indicate that steric fields, depicted in Figure 3S, are primarily concentrated near the two aromatic rings (Ar_1_ and Ar_2_) at the extremities of the more active molecule (C7). Green and yellow contours, representing 80% and 20% contributions, respectively, highlight the molecular regions that either enhance or reduce AChE inhibitory activity. Almost identical to steric effects, electrostatic fields are created in the vicinity of two extreme benzenic rings (Ar_1_ and Ar_2_) as presented in Figure 3E, whereas other molecular regions negatively impact this field. 80% of blue contours represent favorable electrostatic contributions, while the red contours account for 20% of unfavorable effects. In the CoMSIA/SEHDA model, similar to steric interactions, 80% of the hydrophobic fields favor the first aromatic ring (Ar_1_) at the extremity, as shown by the yellow contours in Figure 4H. Unlike that, hydrogen bond donor fields colored in cyan are predominantly distributed at the invested extremity close to Ar_1_ benzene bound to the nitrogen atom, as shown in Figures 4D, while the acceptor of hydrogen bonds’ fields are well-distributed around both Ar_1_ and Ar_2_ rings, as colored in magenta (Figure 4A), where 80% of favorable donor contributions marked in cyan so 80% of favorable acceptor contributions highlighted in magenta. The mapped favorable interaction zones, including steric and electrostatic, hydrophobic, hydrogen bond donor, and acceptor fields from the CoMFA and CoMSIA/SEHDA models, establish a three-dimensional structure-activity relationship (SAR) between the potential for AChE inhibitory activity and the most active derivative (C7), as depicted in Figure 5. These results have significantly contributed to the identification of seven novel pyrazole and benzofuran-based derivative compounds. Their biological activities, expressed in terms of pIC_50_, are predicted using both CoMFA and CoMSIA/SEHDA models, as presented in Table 4.

**TABLE 4:** Predicted pIC_50_ values of novel compounds designed using both CoMFA and CoMSIA/SEHDA models.

N°	Structure	Predicted pIC_50_ using CoMFA model	Predicted pIC_50_ using CoMSIA/SEHDA model
C7	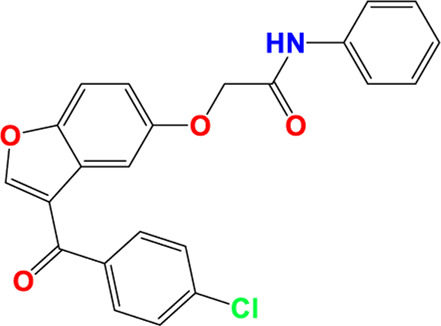	6.297	6.401
27**	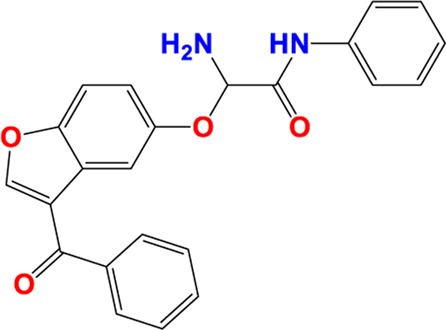	6.132	6.007
28**	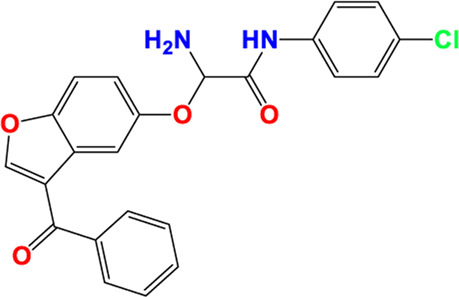	4.735	4.735
31**	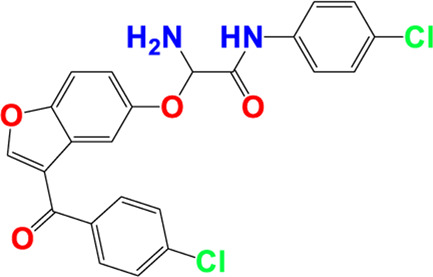	4.841	5.122
32**	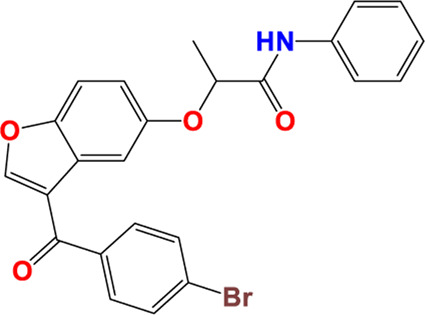	6.019	5.975
34**	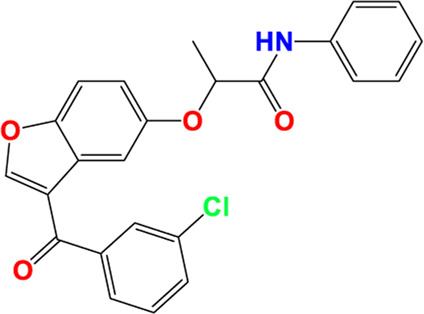	6.109	4.021
35**	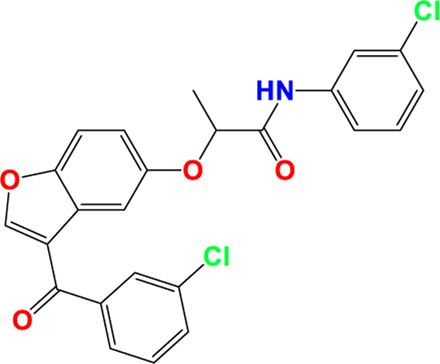	5.819	3.966
37**	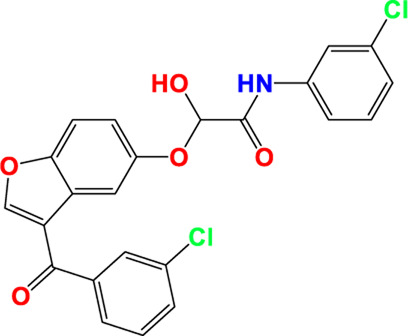	5.901	4.396

### 3.3 Pharmacokinetics profiling

A computational *in silico* assessment of pharmacokinetic properties, including absorption, distribution, metabolism, excretion, and toxicity (ADMET), was performed on newly designed compounds. All the tested compounds demonstrated favorable ADME characteristics, suggesting their potential safety. These molecules exhibited high intestinal absorption rates exceeding 91%, strong permeability across the blood-brain barrier (BBB) and central nervous system (CNS), and potent inhibition of cytochrome enzymes 1A2, 2C9, 2C19, and 3A4. Additionally, the AMES toxicity test confirms the safety of the previously synthesized compound C7, as well as all seven designed compounds C27**, C28**, C31**, C32**, C34**, C35**, and C37**, demonstrating their lack of mutagenic potential and safety from hepatotoxicity and skin sensitization adverse effects, as outlined in Table 5.

**TABLE 5:** ADMET pharmacokinetic features of novel PhO-THA derivatives.

Models	ADME and toxicity properties													
	Absorption	Distribution		Metabolism							Excretion	Toxicity		
	Intestinal absorption (human)	BBB permeability	CNS permeability	CYP450							Total clearance	AMES toxicity	Hepatotoxicity	Skin sensitization
				Substrate		Inhibitor								
				2D6	3A4	1A2	2C19	2C9	2D6	3A4				
Unity	Numeric (%absorbed)	Numeric (Log BB)	Numeric (Log PS)	Categorical (YES/NO)							Numeric (log mL min^−1^kg^−1^)	Categorical (yes/no)		
Predicted values														
C7	90.717	−0.324	−1.794	No	Yes	Yes	Yes	Yes	No	Yes	−0.177	No	No	No
C27**	97.525	−0.467	−2.237	No	Yes	Yes	Yes	Yes	No	Yes	0.783	No	No	No
C28**	98.64	−0.655	−2.126	No	Yes	Yes	Yes	Yes	No	Yes	0.776	No	No	No
C31**	99.821	−0.841	−2.008	No	Yes	Yes	Yes	Yes	No	Yes	0.815	No	No	No
C32**	93.871	−0.419	−1.768	No	Yes	Yes	Yes	Yes	No	Yes	−0.242	No	No	No
C34**	93.772	−0.41	−1.796	No	Yes	Yes	Yes	Yes	No	Yes	−0.105	No	No	No
C35**	92.982	−0.574	−1.682	No	Yes	Yes	Yes	Yes	No	Yes	−0.174	No	No	No
C37**	91.366	−0.794	−2.043	No	Yes	Yes	Yes	Yes	No	Yes	−0.189	No	No	No

To further validate their pharmacokinetic suitability, physicochemical properties were evaluated based on Lipinski’s rule of five along with additional criteria from Egan, Veber, Ghose, and Muegge (Table 6). The results confirmed that all seven compounds met the necessary requirements, including Molecular weight below 500 g/mol, Log P values under 5, Topological surface area less than 140 Å^2^, no more than 10 hydrogen bond acceptors and 5 hydrogen bond donors.

**TABLE 6:** Prediction of physicochemical properties of four synthesized compounds.

Models	TPSA	MW	LogP	n-HBA	n-HBD	Violations number				
						Lipinski	Veber	Egan	Muegge	Ghose
Rule	<140 A^2^	<500 D.a	≤5	<10	<5	≤2	≤2	≤2	≤2	≤2
C7	68.54	405.83	3.08	4	1	Yes	Yes	Yes	No	Yes
C27^a^	94.56	386.40	2.53	5	2	Yes	Yes	Yes	Yes	Yes
C28^a^	94.56	420.85	2.49	5	2	Yes	Yes	Yes	Yes	Yes
C31^a^	94.56	455.29	3.00	5	2	Yes	Yes	Yes	Yes	Yes
C32^a^	68.54	464.31	3.63	7	4	Yes	Yes	Yes	No	No
C34^a^	68.54	419.86	3.71	4	1	Yes	Yes	Yes	No	Yes
C35^a^	68.54	454.30	3.89	4	1	Yes	Yes	No	No	No
C37^a^	88.77	456.27	3.66	5	2	Yes	Yes	Yes	No	Yes

^a^ Indicate novel-designed molecules.

### 3.4 Frontier molecular orbitals analysis

A detailed examination of frontier molecular orbitals (FMOs) offers crucial qualitative insights into the electronic excitation states of a molecule. These states are fundamentally characterized by the energy levels of two primary molecular orbitals: the Highest Occupied Molecular Orbital (HOMO) and the Lowest Unoccupied Molecular Orbital (LUMO) (Montanari et al., 2013). The HOMO energy level represents the molecule’s ability to donate electrons, while the LUMO energy level reflects its capacity to accept electrons, playing a pivotal role in charge transfer phenomena and chemical reactivity (Aloui et al., 2024b). The relative positioning of these orbitals is instrumental in determining the electronic behavior of a molecule, particularly in redox processes, photochemical reactions, and intermolecular interactions.

A key parameter derived from these orbitals is the HOMO-LUMO energy gap (ΔE), which serves as a fundamental indicator of molecular stability and reactivity (Dege et al., 2022; Abdulridha et al., 2020). A smaller ΔE implies a higher propensity for electronic transitions, facilitating charge transfer and enhancing molecular polarizability. Conversely, molecules with a larger HOMO-LUMO gap tend to exhibit greater kinetic stability and reduced reactivity, as electronic excitation requires a higher energy input. This correlation is particularly relevant in the context of chemical hardness and softness, where a lower energy gap is often associated with higher chemical softness, indicating greater reactivity and susceptibility to external perturbations.

To gain a comprehensive understanding of the electronic properties and reactivity of the studied compounds, several quantum chemical descriptors beyond the HOMO and LUMO energies (Aloui et al., 2024a) were computed. These include the ionization energy (I), electron affinity (A), electronegativity (χ), chemical hardness (η), chemical softness (S), electron chemical potential (μ), and the global electrophilicity index (ω). Each of these parameters provides valuable information regarding the stability, charge distribution, and overall reactivity of the molecules. The calculated values for these descriptors are summarized in Table 7, offering a detailed comparative analysis of the electronic characteristics of the studied compounds.

**TABLE 7:** Values obtained during the calculation of chemical reactivity parameters.

N	E_HOMO_	E_LUMO_	∆E	MD	I	A	χ	S	ŋ	µ	ω
C7	−6.166	−1.842	4.324	6.12	6.166	1.842	4.004	1.081	2.162	−4.004	17.334
C27**	−5.849	−1.890	3.959	6.43	5.849	1.890	3.869	0.990	1.979	−3.869	14.818

**FIGURE 6:**
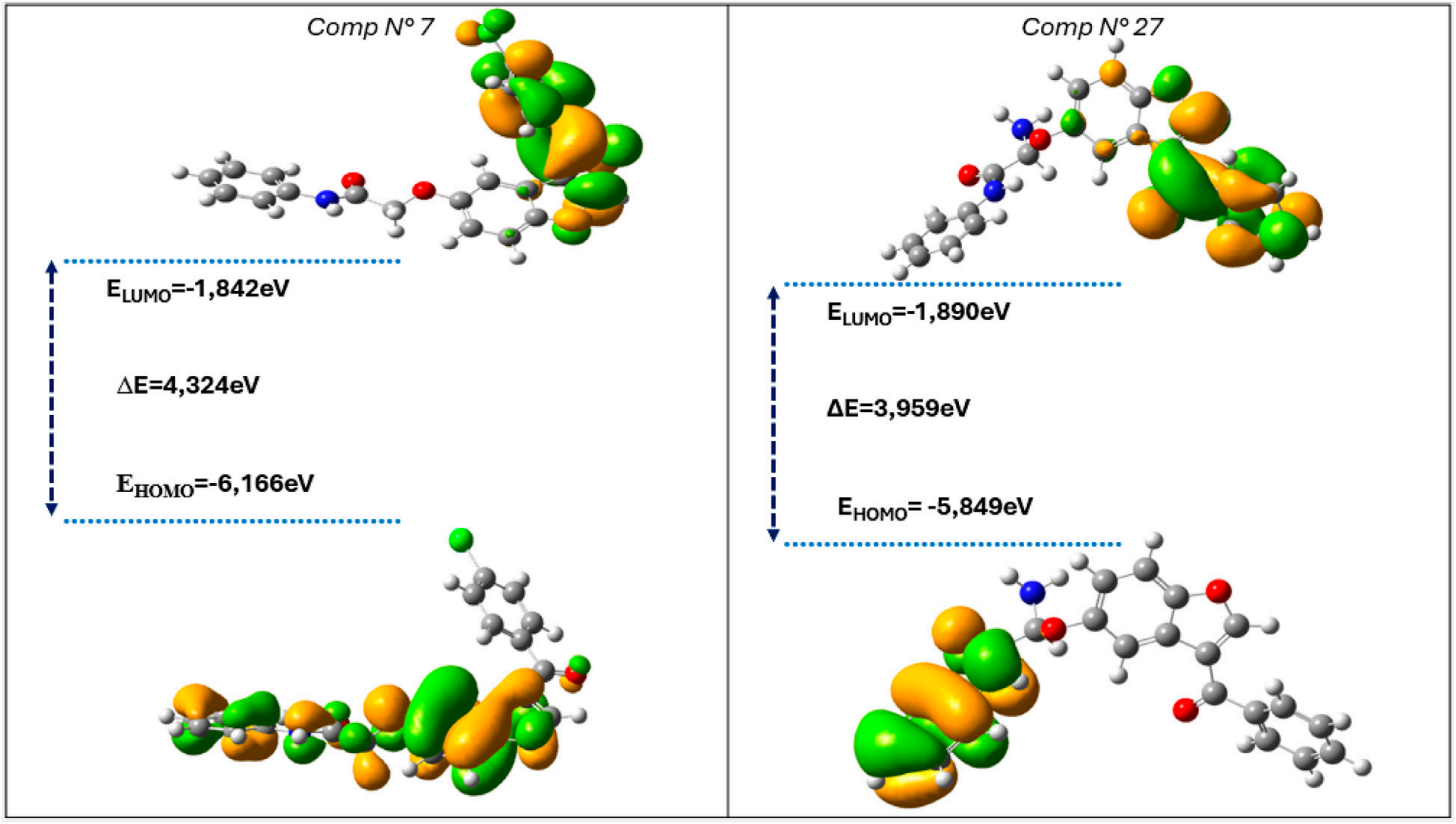
Frontier molecular orbitals of the four molecules studied.

The predicted compound C27** shows lower energy difference (3.959 eV) compared to the most active molecule in the set studied (Reference C7), suggesting higher activity. A smaller difference between the energies of the frontier orbitals (HOMO and LUMO) is generally associated with better bioactivity. Among these compounds, predicted compound C27** displays the lowest energy gap compared to molecule Ref C7, the most active of the set studied, making it a potentially promising candidate as an acetylcholinesterase inhibitor (anti-ACh). Notably, compound C27** has a slightly higher ionization energy (5.849 eV) as well as maximum electron affinity (1.890 eV), indicating a greater ability to capture and give up electrons. Chemical hardness (η) is a key parameter reflecting the stability of a molecule and is directly related to the HOMO-LUMO energy gap. A larger gap between these orbitals indicates greater stability and less reactivity, as higher energy is required to excite an electron and initiate a chemical reaction. Predicted compounds C27** show the lowest chemical hardness value, suggesting that it is less stable and more reactive than the other molecules studied. Chemical electron potential (μ) quantifies the ease with which a molecule can give up or take up electrons. Compound C7 has a higher chemical potential (−4.004 eV and −4.178 eV), implying greater electron flow. In addition, the predicted compounds 27 has higher dipole moment (6.43 D) compared to the most active molecules (Ref C7), which could indicate a stronger interaction with other molecules in their environment. Finally, compound 27**, with the lowest energy gap, also displays a high electronegativity (3.236), confirming its reactive nature. Furthermore, this molecule has a higher electrophilicity index (ω) than the other molecules (Figure 6), suggesting a greater propensity for electron transfer from the HOMO to the LUMO level. This increased reactivity and potentially reduced stability led us to analyze the predicted most active molecules (compound C27**) as well as the most active molecule (C7), using molecular electrostatic potential (MEP). This technique identifies the regions of each molecule likely to donate or accept electrons, thus facilitating the localization of electrophilic and nucleophilic sites.

### 3.5 Analysis of the molecular electrostatic potential

**FIGURE 7:**
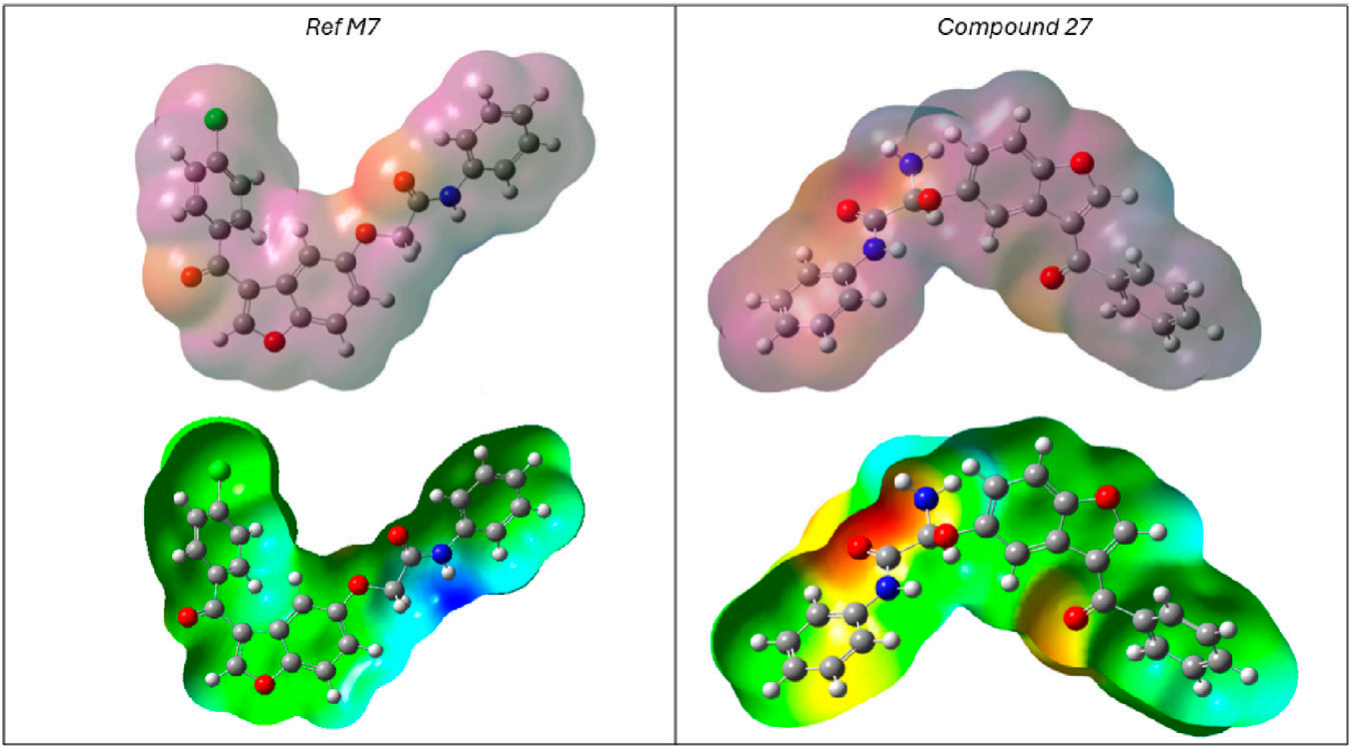
Molecular Electrostatic Potential of the most active predicted molecule (Compound 27) and the most active molecules in the studied set (Ref C7).

The MEP map, a key tool in molecular biology and chemistry, illustrates the electric charge distribution within a molecule, facilitating the study of its interactions with other molecules. The MEP analysis of compounds C27** and reference C7 (Figure 7) provides valuable insights into biological recognition, hydrogen bonding, and electrophilic/nucleophilic reactivity. MEP calculations, performed using the optimized B3LYP/6-31G (d,p) geometry, allow for the analysis of electrostatic potential distribution and the prediction of reactive sites likely to be targeted by electrophilic or nucleophilic species. Molecular electrostatic potential is typically visualized as a color-coded surface mapped onto the molecular structure, with red indicating regions of negative electrostatic potential (electron-rich), blue showing areas of positive potential (electron-deficient), and green to yellow representing near-neutral zones. This visual representation provides insight into the charge distribution within a molecule, helping to identify sites susceptible to electrophilic or nucleophilic attack. Figure 7 shows that the reference compound C7 exhibits a prominent blue region around the hydrogen-bonded nitrogen atoms, indicating areas of low electron density and potential electrophilic reactivity. In contrast, the MEP map of the designed compound C27** reveals a significant red region around the ketone group, suggesting strong nucleophilic character. Additionally, the green to yellow regions in both compounds are predicted to be electrostatically neutral.

### 3.6 Molecular docking simulations

**FIGURE 8:**
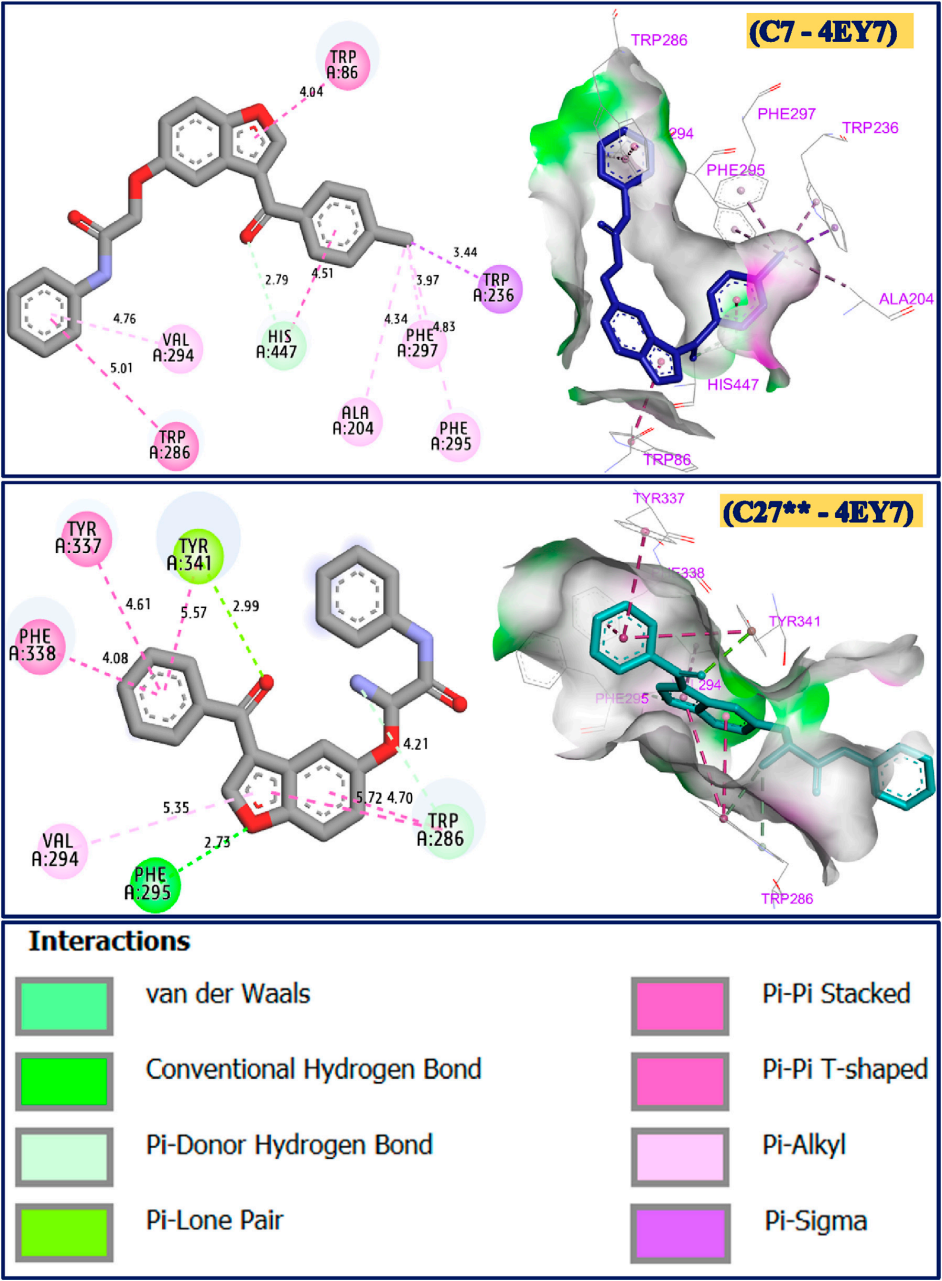
Docking results in two and three dimensions for C7 and C27** chemical compounds during their complexation towards acetylcholinesterase protein (PDB ID of 4EY7).

The molecular docking simulations were carried out for C7 as the synthesized molecule with the strongest AChE inhibitory activity, as well as C27** as the most active designed compound to be complexed against the targeted protein, namely, the crystal structure of human Acetylcholinesterase (PDB ID of 4EY7). The compound C7, colored in blue, was first docked to the crystal structure of recombinant human acetylcholinesterase protein with a binding energy of −9.39 kcal/mol, revealing various chemical interactions, including two Pi-Pi T-shaped bonds fixed to Trp86 and Trp286 amino acid residues, one Pi-sigma bond detected with Trp236, one Pi-Donor hydrogen bond formed with His447, in addition to several alkyl and Pi-alkyl bonds detected with Phe297, Phe295, Ala204 and Val294 amino acids. Secondly, the candidate compound colored in cyan (C27**), which was designed and discovered with the lowest AChE inhibitory activity, was equally docked to the same targeted receptor with a binding energy of −7.88 kcal/mol, revealing almost the same previous interactions, including one conventional hydrogen bond created with the Phe295 amino acid, two Pi-Pi T-shaped bonds’ fixed with Phe338 and Tyr337 amino acids, one Pi-Alkyl bond formed towards Val294 amino acid, one Pi-Lone pair bond detected with Tyr341 amino acid residue, in addition to Pi-Donor hydrogen bond fixed with Trp286 active site, as shown in Figure 8.

### 3.7 Docking validation protocol

**FIGURE 9:**
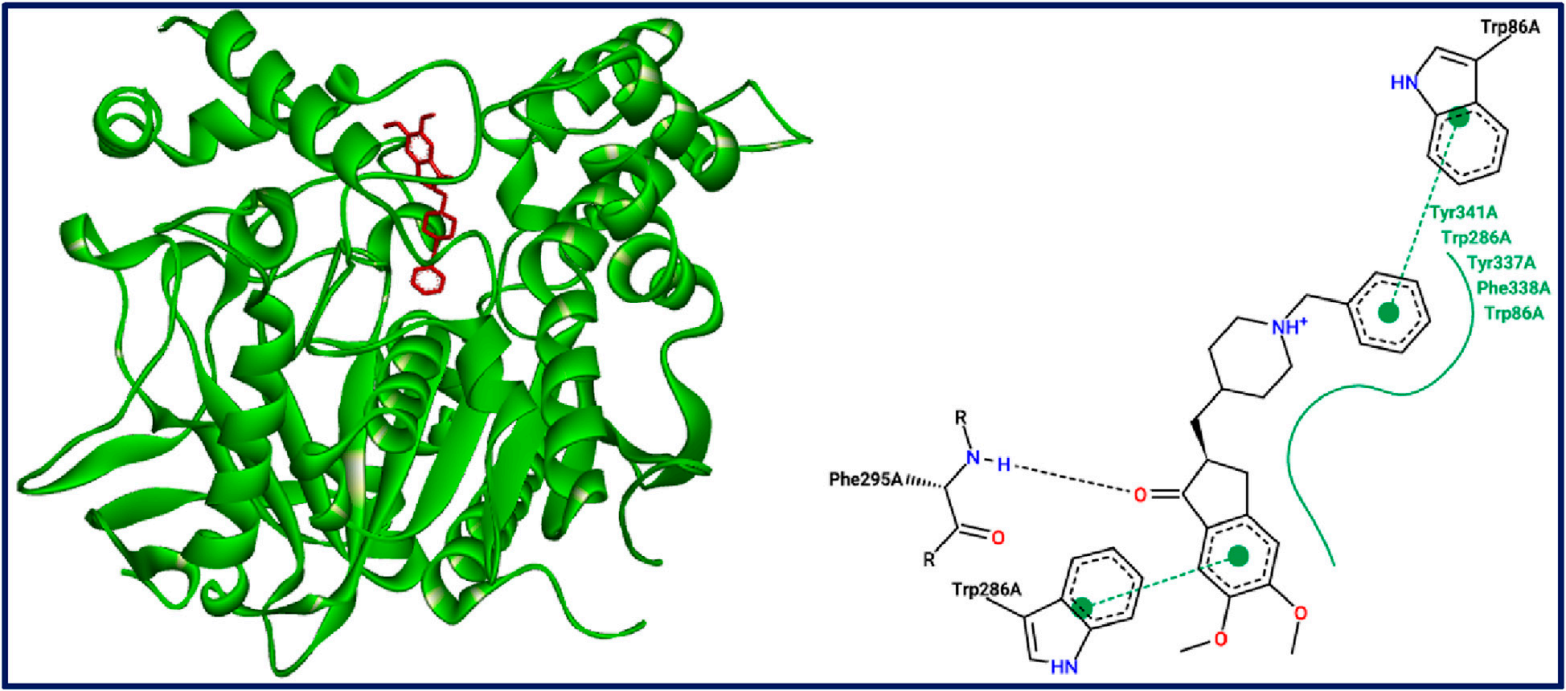
Active sites of the crystal structure of recombinant human acetylcholinesterase in complex with Donepezil drug.

To evaluate the effectiveness of the molecular docking simulations, the intermolecular interactions of the docked compounds C7 and C27** were compared with those observed in the crystal structure of recombinant human acetylcholinesterase (AChE) complexed with its co-crystallized ligand (marked in red in Figure 9). In this reference structure, the drug Donepezil interacts with key active site residues Trp86, Trp286, and Phe295 of the AChE protein’s A chain. For comparison, similar interactions were identified for both examined small molecules in complex with the target receptor, indicating successful docking to the active sites of the target protein. The designed compound (C27**) docked effectively at two active sites (Trp286 and Phe295) in chain A, while the strongest synthesized compound (C7) engaged all three active sites (Trp86, Trp286, and Phe295) in chain A of the AChE protein. These findings demonstrate that both compounds docked close to the active sites of the AChE receptor, confirming the successful validation of the docking protocol.

### 3.8 Molecular dynamics simulations

**FIGURE 10:**
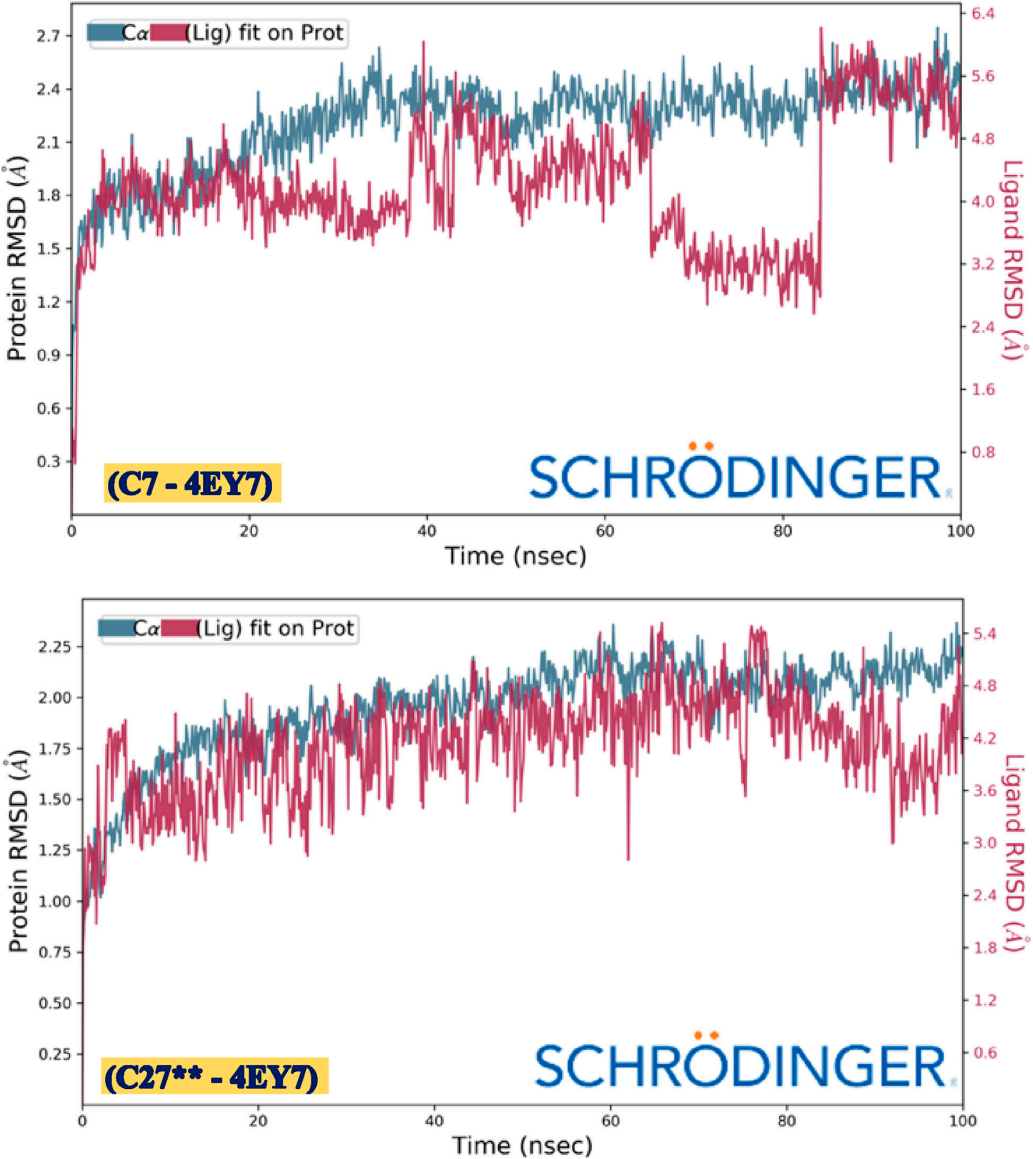
Conformational changes in RMSD values during 100 ns of AChE receptor complexed with ligand C7 and ligand C27**, respectively.

**FIGURE 11:**
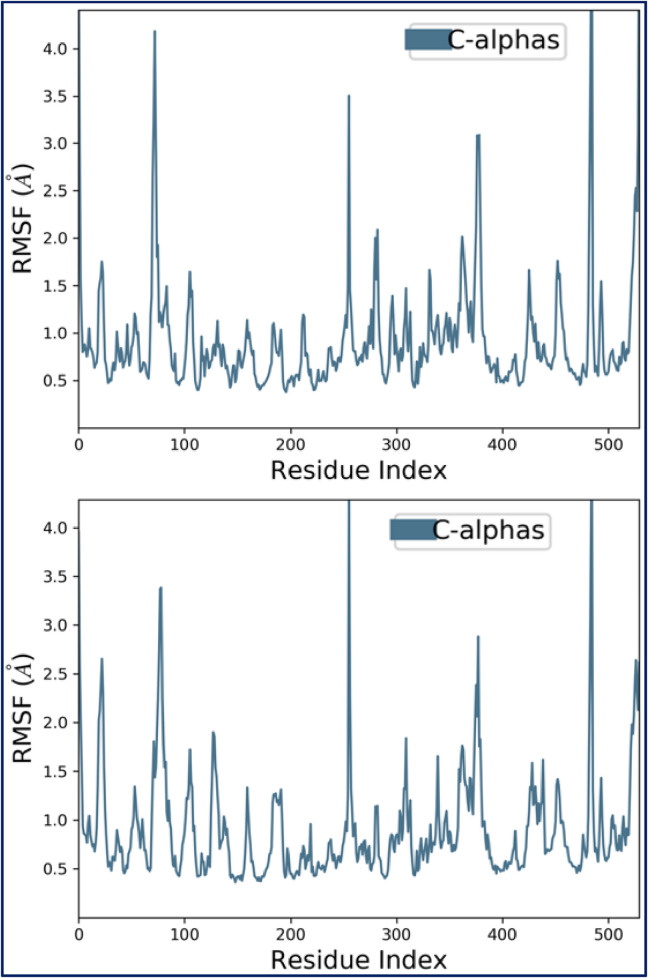
Conformational changes in RMSF values of AChE receptor during its complexation to C7 and C27**, respectively.

MD simulation for 100 ns was executed to validate the thermodynamic stability at the molecular level based upon their intermolecular interactions observed via molecular docking for both the designed pyrazole-based ligands C27** and C7 complexed with the AChE enzyme. Free energy landscape (FEL) analysis was performed for the MD simulation outcomes for each of the considered macromolecular complex by considering root mean square deviations (RMSD), root mean square fluctuations (RMSF), principal component analysis (PCA), solvent accessible surface area (SASA), etc. confirmed an excellent level of molecular stability for both tested compounds in complex with the target receptor. The thermodynamic stability of both two concerned macromolecular complexes of pyrazole-based designed ligands C27** and C7 complexed with AChE enzyme was justified by minimal conformational changes that oscillate around the desired equilibrium without exceeding the 3Å threshold throughout the 100 ns of MD simulation time. The RMSD graph generated for the macromolecular complexes post MD simulation of 100 ns have revealed that the macromolecular complex of AChE receptor with designed compound C27** was showing high stability without any fluctuations with an RMSD value in the range of 1.3–2.1 Å for the Cα backbone of the target AChE receptor while the complexed ligand C27** has shown in the range of 3.8–5.2 Å. The RMSD value observed for the macromolecular complex of AChE receptor with designed compound C7 has revealed that RMSD value observed for the Cα backbone of the target AChE receptor was within the range of 1.6–2.4 Å, while the complexed ligand C7 has taken couple of initial moves for their adjustment within the macromolecular cavity to achieve most stabilized conformation post 80 ns of the MD simulation with the RMSD value ranging between 3.2–5.6 Å. The RMSD changes observed for the two concerned macromolecular complexes are depicted in Figure 10. Additionally, nearly identical conformational changes were detected in the RMSF frequencies of both the complexes of AChE receptors after complexation with both candidate ligands (C7 and C27**) within the range of 0.5–1.5 Å for their Cα backbone, indicating their high stability throughout the simulation. The RMSF graphs for the target receptors were displayed in Figure 11.

**FIGURE 12:**
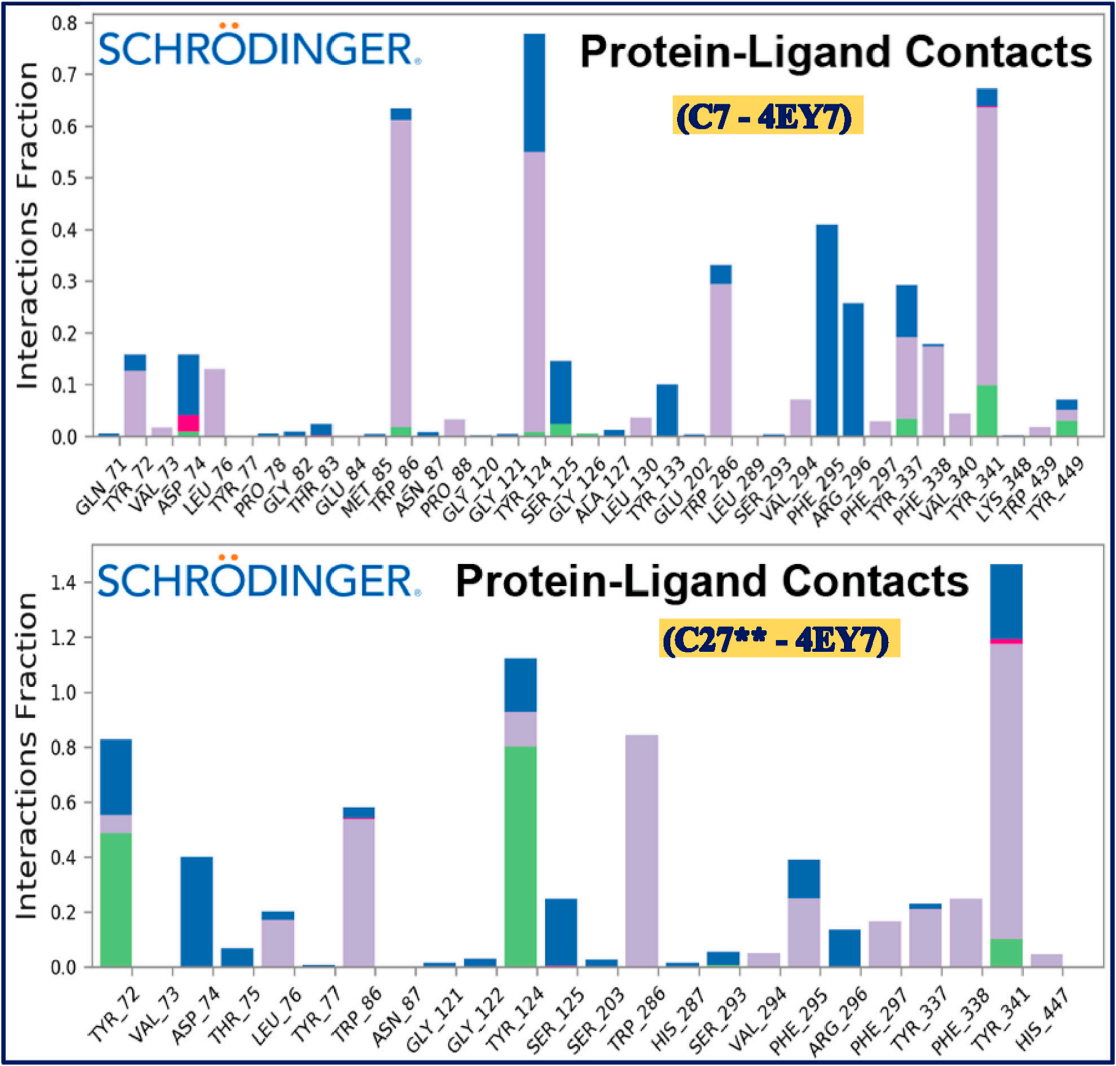
Protein-ligand contacts observed after 100 ns MD simulation for the AChE receptor complexed with ligand C7 and ligand C27**, respectively.

Furthermore, the histogram of protein-ligand contacts generated after MD simulation for all the concerned macromolecular complexes revealed the intermolecular interactions existing between the ligand and the receptor molecule. Ligand C7 was found to be interacting with human AChE receptor via formation of hydrophobic bonds with the amino acids Tyr72, Leu76, Trp86, Tyr124, Trp286, Val294, Tyr337, Phe338, Tyr341, whereas Trp86, Tyr337, Tyr341, and Tyr449 via hydrogen bonds and amino acid Asp74, Tyr124, Ser125, Tyr133, Phe295, Arg296 and Tyr337 are found to be interacting via water bridges. Ligand C27** was found to be interacting with human AChE receptor via formation of hydrophobic bonds with the amino acids Leu76, Trp86, Tyr124, Trp286, Val294, Phe295, Phe297, Tyr337, Phe338, Tyr341, His447 whereas Tyr72, Tyr124, and Tyr341 via hydrogen bonds and amino acid Tyr72, Asp74, Thr75, Ser125, Ser293, Phe295, Arg296 and Tyr341 are found to be interacting via water bridges. The MD simulation results confirm that intermolecular interactions produced by molecular docking are a key function in achieving the thermodynamic stability of the macromolecular complex. Figure 12 depicts the observed protein ligand contacts for the AChE receptor complexed with ligand C7 (a) and ligand C27** (b), respectively.

## 4 Conclusion

In conclusion, seven novel and safe pyrazole and benzofuran derivatives were successfully designed through a three-dimensional QSAR investigation as potent acetylcholinesterase (AChE) inhibitors, exhibiting favorable physicochemical and ADMET pharmacokinetic profiles. The CoMFA and CoMSIA/SEHDA models revealed that both the newly designed compound C27** and the previously synthesized compound C7 showed enhanced predicted anti-AChE activity. The candidate compounds demonstrated excellent thermodynamic stability within the active site of the AChE receptor (PDB ID: 4EY7) during 100 nanoseconds of molecular dynamics simulation, indicating strong potential as anti-Alzheimer’s drug candidates with high structural and functional similarity to existing therapeutics. However, further experimental validation and clinical trials are essential to confirm their efficacy and safety for the human body.

## Data Availability

The original contributions presented in the study are included in the article/supplementary material, further inquiries can be directed to the corresponding authors.
